# Fabrication of Hybrid Nanostructures Based on Fe_3_O_4_ Nanoclusters as Theranostic Agents for Magnetic Resonance Imaging and Drug Delivery

**DOI:** 10.1186/s11671-019-3026-7

**Published:** 2019-06-07

**Authors:** Junwei Zhao, Xiang Li, Xin Wang, Xin Wang

**Affiliations:** 1 Henan Key Laboratory of Photovoltaic Materials, Henan University, Kaifeng, 475004 People’s Republic of China; 2 Materials Science and Engineering School & Henan Key Laboratory of Special Protective Materials, Luoyang Institute of Science and Technology, Luoyang, 471023 People’s Republic of China; 3 College of Materials Science and Engineering, Jilin University, Changchun, 130022 People’s Republic of China; 4 Division of Nanobiomedicine, Suzhou Institute of Nano-Tech and Nano-Bionics, Chinese Academy of Sciences, Suzhou, 215123 People’s Republic of China

**Keywords:** Fe_3_O_4_, Nanoclusters, DOX, Self-assembly, Hybrid nanostructures, Drug delivery

## Abstract

Combining anticancer drugs with inorganic nanocrystals to construct multifunctional hybrid nanostructures has become a powerful tool for cancer treatment and tumor suppression. However, it remains a critical challenge to synthesize compact, multifunctional nanostructures with improved functionality and reproducibility. In this study, we report the fabrication of magnetite hybrid nanostructures employing Fe_3_O_4_ nanoparticles (NPs) to form multifunctional magnetite nanoclusters (NCs) by combining an oil-in-water microemulsion assembly and a layer-by-layer (LBL) method. The Fe_3_O_4_ NCs were firstly prepared via a microemulsion self-assembly technique. Then, polyelectrolyte layers composed of poly(allylamine hydrochloride) (PAH) and poly(sodium 4-styrenesulfonate) (PSS) and doxorubicin hydrochloride (DOX) were capped on Fe_3_O_4_ NCs to construct the Fe_3_O_4_ NC/PAH/PSS/DOX hybrid nanostructures via LBL method. The as-prepared hybrid nanostructures loaded with DOX demonstrated the pH-responsive drug release and higher cytotoxicity towards human lung cancer (A549) cells in vitro and can serve as *T*_2_-weighted magnetic resonance imaging (MRI) contrast agents, which can significantly improve *T*_2_ relaxivity and lead to a better cellular MRI contrast effect. The loaded DOX emitting red signals under excitation with 490 nm are suitable for bioimaging applications. This work provides a novel strategy to build a Fe_3_O_4_-based multifunctional theranostic nanoplatform with *T*_2_-weighted MRI, fluorescence imaging, and drug delivery.

**Electronic supplementary material:**

The online version of this article (10.1186/s11671-019-3026-7) contains supplementary material, which is available to authorized users.

## Introduction

In recent years, various multifunctional drug delivery systems have been developed for future diagnosis and therapy in biomedical application [1–4]. Multifunctional hybrid nanostructures that are integrated favorable properties will possess significant applications such as multimodal imaging and simultaneous diagnosis and therapy [5–11]. Furthermore, these nanostructures are stimuli-responsive drug delivery systems for improved drug accumulation, enhanced therapeutic efficacy, and/or reduced side effects. Especially, these pH-responsive drug delivery systems have attracted extensive research interest. This is because most human tumors have a more acidic pH value, which provides a possible way to design the controlled release of drug molecules [12–16].

Over the past few decades, various hybrid nanostructures by combining inorganic nanomaterials with organic polymer [17–20] have been developed, including magnetic particles [21–23], upconversion nanoparticles (NPs) [17, 24], and mesoporous silica particles [25]. Among those, magnetic hybrid nanostructures based on iron oxides with relatively large magnetization at room temperature have been widely used in the biomedical fields [26–29]. The functionalization of inorganic nanomaterials coated with polyelectrolyte layers can realize a pH-responsive encapsulation and release of drug molecules [12, 17, 30]. More recently, the polyelectrolyte layers composed of sodium poly (styrene sulfonate) (PSS) and the polycation poly(allylamine hydrochloride) (PAH) has been widely studied [31–36]. Polyelectrolyte layers combined with magnetic and luminescent NPs or drug molecules for multifunctional drug delivery systems have also been recently reported [37–39]. Iron oxide (Fe_3_O_4_) NPs are getting more and more attention in the field of magnetic resonance imaging (MRI) and drug delivery due to their unique superparamagnetic properties, biocompatibility, low-cytotoxicity, and flexibility [9, 11, 28, 29, 40–42]. In general, there are two methods to improve the magnetic responsiveness of Fe_3_O_4_ NPs. The first one is to synthesize the micrometer-sized magnetite particles. Due to large size, however, they tend to aggregate in aqueous solution, which is not beneficial to biomedical applications. The other approach is to assemble Fe_3_O_4_ NPs into nanoclusters (NCs). These Fe_3_O_4_ NCs greatly improved the magnetic responsiveness compared to individual Fe_3_O_4_ NPs [22, 43]. Therefore, if the self-assembled Fe_3_O_4_ NCs are adopted as the core to fabricate multifunctional hybrid nanostructures, the MRI performance will be improved by the collective effect of Fe_3_O_4_ NPs [43–45]. To our knowledge, the self-assembled Fe_3_O_4_ NCs functionalized with PAH/PSS multilayers for pH-responsive drug release have rarely been reported.

In this work, a versatile theranostic nanoplatform based on Fe_3_O_4_ NPs was built up for MRI and drug delivery. In our approach, Fe_3_O_4_ NC/PAH/PSS/DOX hybrid nanostructures were obtained by combining an oil-in-water microemulsion method and a layer-by-layer (LBL) electrostatic adsorption method. It is expected that the packed Fe_3_O_4_ NC system can lead to enhanced *T*_2_ relaxivity and imaging contrast, and the large specific surface area of Fe_3_O_4_ NC/PAH/PSS hybrid nanostructures allows high loading of anticancer drugs. Moreover, in vitro experiment exhibits that the cellular MRI contrast of human lung cancer (A549) cells incubated with Fe_3_O_4_ NC/PAH/PSS/DOX has been significantly enhanced.

## Materials and Methods

### Materials

FeCl_3_·6H_2_O (99.99%), FeCl_2_·4H_2_O (99.99%), oleic acid (OA, 90%), and 1-octadecene (ODE, 90%) were purchased from Alfa Aeasar. Sodium oleate (NaOA), ethanol, hexane, cyclohexane, isopropanol, sodium dodecyl benzene sulfonate (SDBS), ammonium fluoride (NH_4_F), sodium hydroxide (NaOH), dimethyl sulfoxide (DMSO), and ammonia were purchased from Sinopharm Chemical Reagent Co., Ltd (China). Poly(allylamine hydrochloride) (PAH), poly(styrene sulfonate) (PSS), and 3-(4,5-dimethylthiazol-2-yl)-2,5-diphenyltetrazolium bromide were purchased from Sigma-Aldrich. Anticancer drug doxorubicin hydrochloride (DOX, > 98%) was purchased from Shanghai Sangon Biotech Company (Shanghai, China). APMI 1640 growth medium and fetal bovine serum (FBS) were purchased from Hyclone. All reagents were used as received without further purification.

### Preparation of Ferric Oleate

The synthesis of magnetic NPs started from the synthesis of ferric oleate. FeCl_3_·6H_2_O (2.59 g), NaOA (14.6 g), C_2_H_5_OH (32 mL), H_2_O (24 mL), and hexane (56 mL) were mixed together in a 150-mL three-neck flask and heated to 70 °C for reflux for 4 h to form a transparent ferric oleate complexes solution. After that, the liquid was separated by a separation funnel and the upper oil layer was preserved. Hexane in the liquid evaporated at 70 °C by rotating evaporation and dried for 48 h under vacuum. The prepared samples were stored in a vacuum glove box for further use.

### Synthesis of Fe_3_O_4_ NPs

We synthesized Fe_3_O_4_ NPs following previously reported procedures with slight modification [46]. Ferric oleate (7.2 g), OA (1.28 mL), and ODE (50 mL) were mixed together in a 100-mL three-neck flask and heated to 300 °C for 40 min under argon protection; after that, the mixture was cooled to room temperature and oxidized in air for more than 12 h. The resultant nanocrystals were precipitated by the addition of isopropanol, centrifuged, and washed twice with an ethanol–water mixture (1:1 *v*/*v*). The oleic acid-capped Fe_3_O_4_ NPs were finally dispersed in 200 mL cyclohexane, and the supernatant was sealed and stored for the subsequent experiments.

### Preparation of Fe_3_O_4_ NCs

Fe_3_O_4_ NCs were prepared by a facile and straightforward microemulsion self-assembly technique as previously described with modification [47]. Briefly, a 200-μL solution of Fe_3_O_4_ nanocrystals in cyclohexane was poured into 4 mL of aqueous solution containing 14 mg of SDBS. The mixed solution underwent ultrasonic treatment for 5 min for 4 times. The formed solid-in-oil-in-water (S/O/W) emulsion was stirred at room temperature for 6 h to evaporate the organic solvent following by the self-assembly of Fe_3_O_4_ NPs to form 3D NCs. The final products were washed with deionized water 3 times to remove the excess SDBS, unincorporated nanocrystals, and some possible larger contaminants.

### Preparation of Fe_3_O_4_ NC/PAH/PSS/DOX Hybrid Nanostructures

The Fe_3_O_4_ NC/PAH/PSS/DOX hybrid nanostructures were prepared by electrostatic attractive interactions. The as-prepared Fe_3_O_4_ NCs are negatively charged owing to the encapsulation of the anionic surfactants. They were first turned to be positively charged by adsorption of a layer of positively charged polyelectrolyte, poly(allylamine hydrochloride) (PAH, MW 15 000). Specifically, a 300-μL Fe_3_O_4_ NC sample was firstly diluted 10 times to 3 mL using deionized water. The Fe_3_O_4_ NC mixture was subsequently added dropwise to an aqueous PAH solution (1 mL, 10 g/L, 4 mM NaCl) under vigorous stirring. After the solution was stirred for 24 h, the excess PAH was removed by centrifugation, and the resultant PAH-coated Fe_3_O_4_ NCs (Fe_3_O_4_ NC/PAH) were redispersed in water (3 mL).

The Fe_3_O_4_ NC/PAH were then turned to be negatively charged by adsorption of a layer of negatively charged polyelectrolyte, poly-(sodium 4-styrenesulfonate) (PSS, MW 70 000). Specifically, a 3-mL Fe_3_O_4_ NC/PAH sample solution was added dropwise to an aqueous PSS solution (1 mL, 10 g/L, 4 mM NaCl) under vigorous stirring. After the solution was stirred for 24 h, the excess PSS was removed by centrifugation, and the resultant PSS-coated Fe_3_O_4_ NC/PAH (Fe_3_O_4_ NC/PAH/PSS) were redispersed in water (3 mL).

The DOX aqueous stock solution was first prepared [17]. The concentration was 5.0 mg/mL. The hybrid nanostructure solution was obtained by mixing the Fe_3_O_4_ NC/PAH/PSS solution (3 mL, 32 mg/mL) and the stock DOX solution (60 μL) in a small plastic tube with stirring for 24 h in the darkroom. After centrifugation, the Fe_3_O_4_ NC/PAH/PSS/DOX hybrid nanostructures were obtained finally.

### MRI Measurements

The MRI measurements were performed in an 11.7 T micro 2.5 micro-imaging system (Bruker, Germany). The different amount of the Fe_3_O_4_ NC/PAH/PSS/DOX hybrid nanostructures were dispersed in 1.2 mL agarose aqueous solution and then loaded into the microtubes for MRI measurements. The final Fe ion concentration were 0 mM, 0.013 mM, 0.026 mM, 0.032 mM, 0.041 mM, 0.052 mM, and 0.065 mM, respectively. The measurement parameters are as follows: repetition time (TR) = 300 ms, echo time (TE) = 4.5 ms, imaging matrix = 128 × 128, slice thickness = 1.2 mm, field of view (FOV) = 2.0 × 2.0 cm, and number of averages (NA) = 2.

### Cellular Uptake and MR Imaging

To demonstrate efficient cellular uptake, the A549 cells were seeded on the coverslip in the confocal dish and incubated in a humidified 5% CO_2_ atmosphere for 4 h at 37 °C. Then, the Fe_3_O_4_ NC/PAH/PSS/DOX hybrid nanostructures were added into the incubation medium at the different concentration and incubated for 2 h. The final Fe ion concentrations were obtained as 0, 2.2, 4.5, 9.0, and 13.5 μM, respectively. After the medium was removed, the cells were washed twice with PBS (pH = 7.4, 20 mM) and directly used for MR imaging.

### Standard Curve of DOX

A suitable quantity of DOX was dissolved in water by oscillation. Then, a series of different concentrations of DOX aqueous solution were prepared (0–0.03 mg/mL). The fluorescence intensity of different concentrations of DOX solution was measured (*λ*_ex_ = 490 nm). Finally, the standard curve of DOX was determined through the curve fitting of the fluorescence intensity *vs* the DOX concentration.

The area standard curve: *Y* = 447.4423 + 69745.08457X.

Precision rate of standard curve: *R*^2^ = 0.9992.

### DOX Loading and Release

To measure the loading capacities of the Fe_3_O_4_ NC/PAH/PSS/DOX hybrid nanostructures, the supernatant solution was collected after centrifugation of the as-prepared hybrid nanostructures. The fluorescence spectrum of DOX molecules in the supernatant solution was examined and the concentration of DOX in the supernatant was calculated by comparing the standard curve of DOX. The percentages of DOX remaining in the Fe_3_O_4_ NC/PAH/PSS/DOX hybrid nanostructures were calculated according to the following equation:

$$ \mathrm{Loading}\ \mathrm{efficiency}\ \left(\%\right)=\left({\mathrm{W}}_0\hbox{-} {\mathrm{W}}_{\mathrm{s}}\right)/{\mathrm{W}}_0\times 100\% $$

where *W*_0_ and *W*_s_ represent the initial DOX mass and the DOX mass in the supernatants, respectively.

For the cumulative DOX release studies in PBS buffer solutions (pH 5.0 and 7.4) with the same NaCl concentration of 0.15 M, the Fe_3_O_4_ NC/PAH/PSS/DOX hybrid nanostructures were dispersed in 1.0 mL of buffer solution and then transferred into a dialysis bag. Then, it was kept in buffer solution and gently shaken at 37 °C in the darkroom. At selected time intervals, 100 μL of solution was withdrawn and analyzed by fluorescence spectrum, and then returned to the original solution.

### In Vitro Cytotoxicity of Fe_3_O_4_ NC/PAH/PSS/DOX Hybrid Nanostructures

In vitro cytotoxicity of the Fe_3_O_4_ NC/PAH/PSS/DOX hybrid nanostructures was assessed against A549 cells based on the standard methyl thiazolyltetrazolium (MTT) assay. A549 cells were cultured in APMI 1640 growth medium complemented with 10% fetal bovine serum (FBS), streptomycin at 100 μg/mL, and penicillin at 100 μg/mL. The cells were maintained at 37 °C in a humidified atmosphere of 5% CO_2_ in air. The assay was performed in triplicate with the same manner. Briefly, A549 cells were seeded into 96-well plates at a density of 8 × 10^3^ cells per wells in 100 μL of media. After overnight growth, the cells were then incubated at various concentrations of free DOX, Fe_3_O_4_ NC/PAH/PSS, and Fe_3_O_4_ NC/PAH/PSS/DOX hybrid nanostructures (0.1, 0.2, 0.4, 0.8, 1.2, 1.6, 2.0 μM) for 24 h. After being incubated for 24 h, the 10 μL 3-(4, 5-dimethylthiazol-2-yl)-2,5-diphenyltetrazolium bromide solution (5 mg/mL) was then added each well and the cells were further incubated for 4 h at 37 °C. After the 3-(4, 5-dimethylthiazol-2-yl)-2,5-diphenyltetrazolium bromide solution was removed, 150 μL of dimethyl sulfoxide (DMSO) was added to each well and the plate was gently shaken for 10 min to dissolve the precipitated violet crystals. The optical density (OD) was measured at 490 nm using microplate reader (Perkin Elmer, Victor X4). Cell viability was evaluated as a percentage compared to control cells.

### Characterization

The sizes and morphologies of Fe_3_O_4_ NPs and Fe_3_O_4_ NC/PAH/PSS/DOX hybrid nanostructures were examined by a FEI Tecnai G2-F20 transmission electron microscope (TEM) at an accelerating voltage of 200 kV. Dynamic light scattering (DLS) measurements were performed on a particle size and zeta potential analyzer from Malvern (Zetasizer Nano ZS90). The UV–vis absorption spectra were acquired by a Perkin Elmer Lambda-25 UV–vis spectrometer. The fluorescence spectra were recorded using a Hitachi F-4600 fluorescence spectrophotometer. Inductively coupled plasma atomic emission spectroscopy (ICP-AES) (Agilent 5100) was used to analyze the element Fe concentrations in the Fe_3_O_4_ NC/PAH/PSS/DOX hybrid nanostructures.

## Results and Discussion

**Fig. 1:**
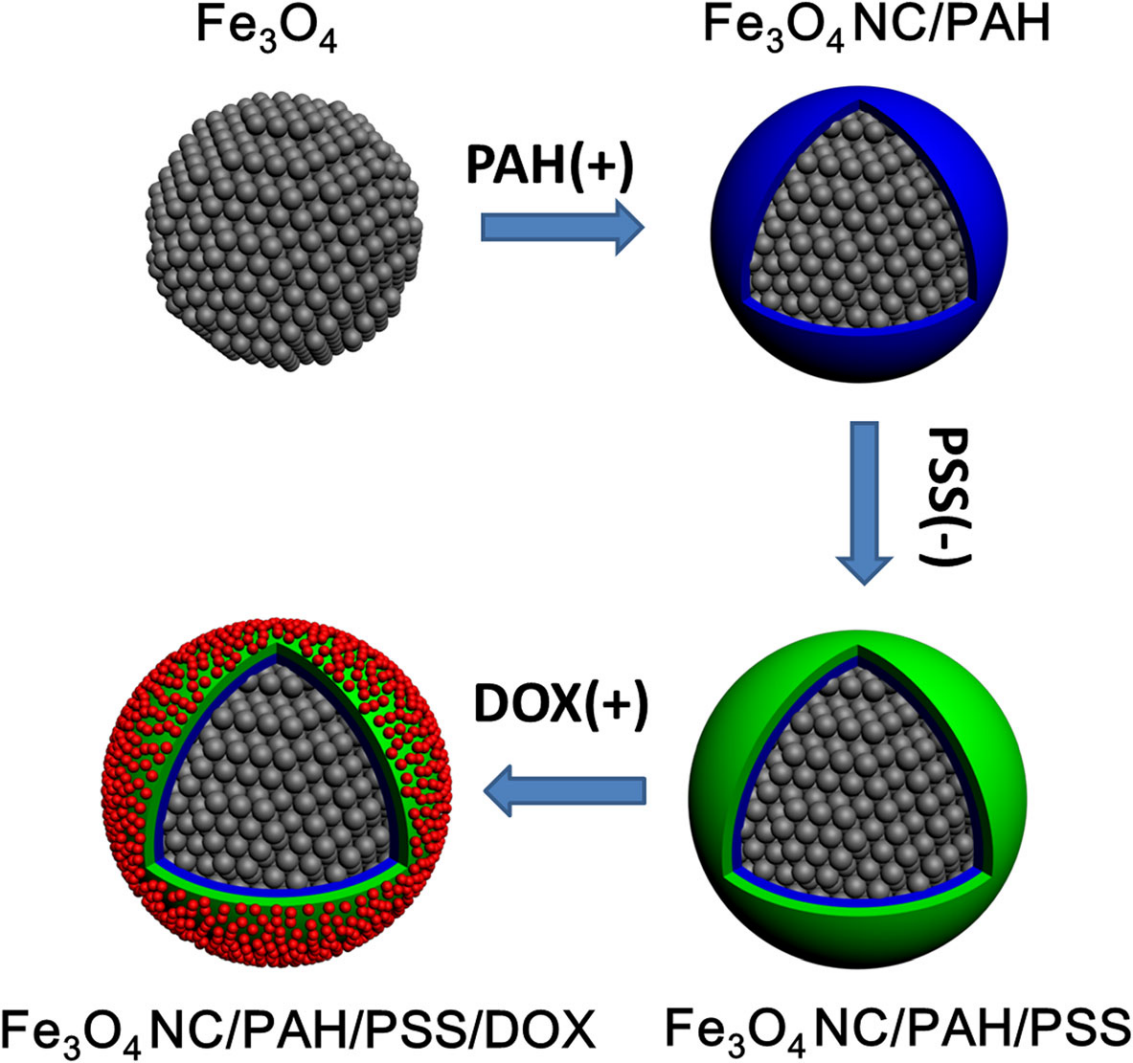
Schematic illustration of synthesis of Fe_3_O_4_ NC/PAH/PSS/DOX hybrid nanostructures as theranostic agents for MRI and drug delivery

The Fe_3_O_4_ NC/PAH/PSS/DOX hybrid nanostructures are prepared by self-assembly of primary iron oxide (Fe_3_O_4_) NPs resulting into densely packed spherical aggregates through a microemulsion self-assembly technique as previously described with modification [17, 47], following by a LBL electrostatic adsorption method. Figure 1 illustrates the schematic illustration of synthesis of Fe_3_O_4_ NC/PAH/PSS/DOX hybrid nanostructures. Hydrophobic oleic acid-coated magnetite Fe_3_O_4_ NPs were initially produced by the thermal decomposition process in organic solvent [46]. Fe_3_O_4_ NPs are spherical and uniform in size with an average particle size of about 15 nm (Additional file 1: Figure S1). For the assembly of magnetic NCs, the OA-coated Fe_3_O_4_ NPs were dispersed in cyclohexane and then added dropwise to an aqueous solution containing SDBS. The complex solution was ultrasonically treated to form a stable oil-in-water emulsion. After the evaporation of organic solvent in the emulsion, Fe_3_O_4_ NPs were self-assembled to form spherical nanoclusters via hydrophobic interaction. Next, the Fe_3_O_4_ NC/PAH/PSS/DOX hybrid nanostructures were prepared via a LBL method through electrostatic attractive interactions, which is schematically illustrated in Fig. 1.

**Fig. 2:**
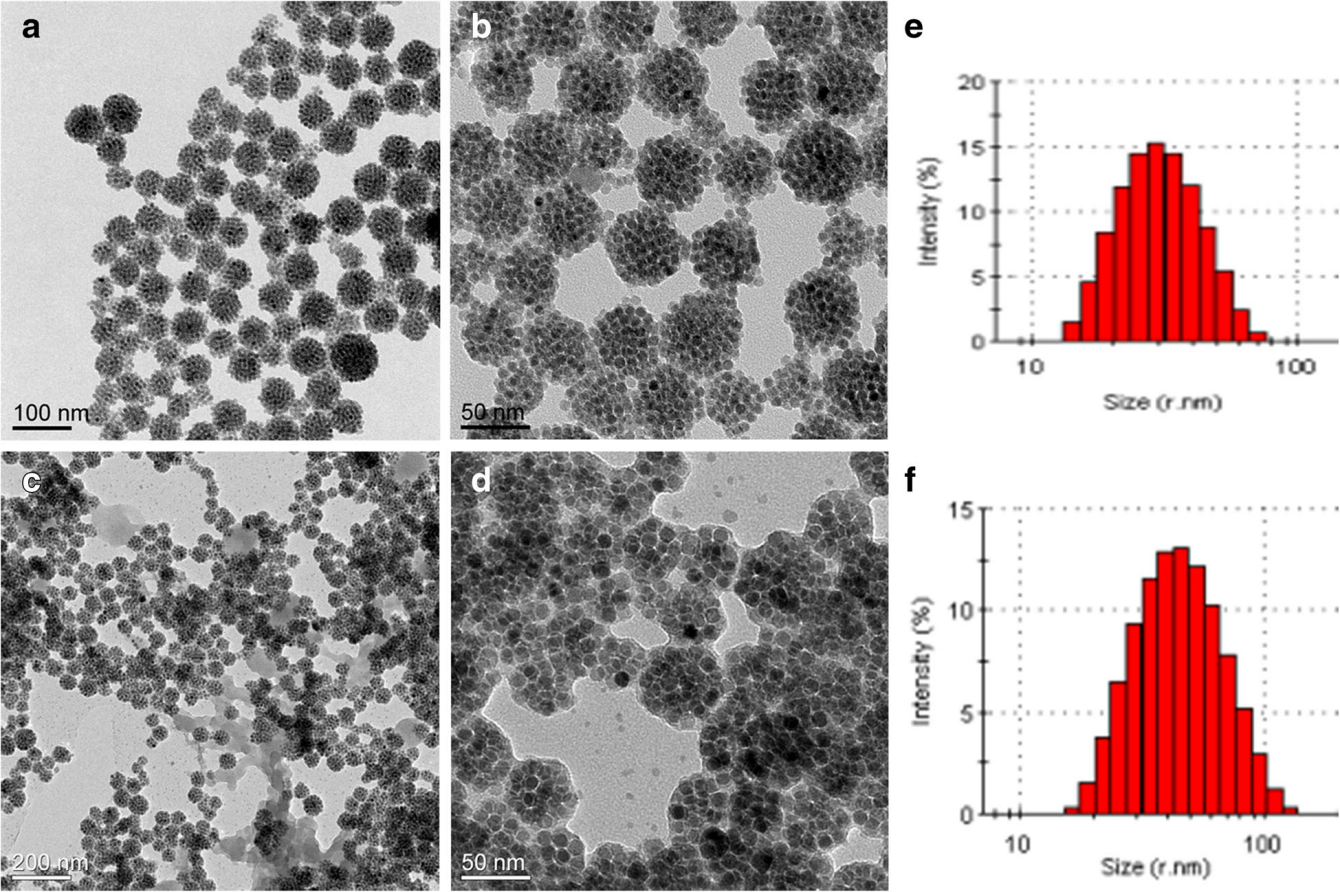
TEM images of Fe_3_O_4_ NCs (**a**, **b**) and Fe_3_O_4_ NC/PAH/PSS (**c**, **d**) at lower and higher magnification, respectively. Size distribution of Fe_3_O_4_ NCs (**e**) and Fe_3_O_4_ NC/PAH/PSS hybrid nanostructures (**f**)

The morphologies and sizes of the Fe_3_O_4_ NCs and the Fe_3_O_4_ NC/PAH/PSS hybrid nanostructures were examined with TEM and DLS, respectively. As shown in Fig. 2a and b, the Fe_3_O_4_ NCs demonstrate the quasi-spherical clusters. The average particle size measured by DLS is about 57 nm (Fig. 2e). In previous reports, PAH with positive charge or PSS with negative charge is alternately deposited on the template surface due to their excellent electrostatic properties [48–51]. To study the formation of each polyelectrolyte layer deposited on the Fe_3_O_4_ NCs, the zeta potential experiments were performed. The variation of zeta potential with the polyelectrolyte layer for PSS/PAH and DOX coatings are shown in Additional file 1: Figure S2. The pristine Fe_3_O_4_ NCs have a negative zeta-potential of − 19.7 mV due to the existence of SDBS. The absorption of a positively charged PAH single layer on Fe_3_O_4_ NCs reverses the surface potential from − 19.7 to + 32 mV. Subsequently, deposition of the negatively charged PSS layer causes another surface potential reversion from + 32 to − 34 mV. This indicates a stepwise layer growth during the fabrication of the magnetic NC hybrid nanostructures. These results show that the PAH and PSS layers were successfully coated on the Fe_3_O_4_ NCs. Finally, DOX was successfully adsorbed on the surface of the Fe_3_O_4_ NC/PAH/PSS/DOX hybrid nanostructures, which was confirmed by the positive zeta potential (+ 1.91 mV) (Additional file 1: Figure S2). The TEM images with a different magnification of the Fe_3_O_4_ NC/PAH/PSS hybrid nanostructures are shown in Fig. 2c and d. No significant structural and morphology difference is observed after polyelectrolyte coatings. Compared with Fig. 2a and b, the bright contrast can be observed and the size of the Fe_3_O_4_ NC/PAH/PSS hybrid nanostructures is increased slightly owing to the coating of PAH and PSS layers. The synthesized magnetic hybrid nanostructures show a nearly mono-dispersed quasi-spherical shape with average size of about 84 nm according to the results of DLS measurement (Fig. 2f).

**Fig. 3:**
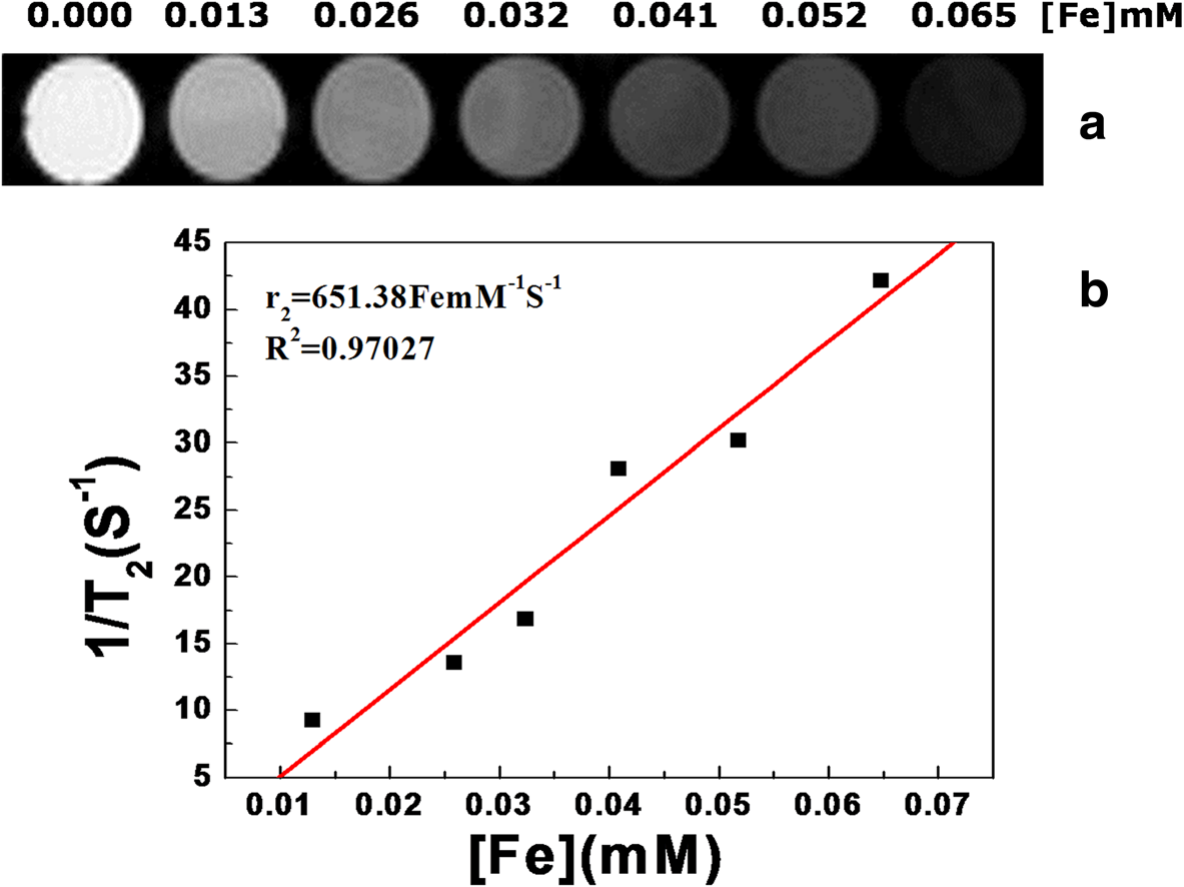
**a** *T*_2_-weighted MRI images of the Fe_3_O_4_ NC/PAH/PSS hybrid nanostructures at different concentrations in water. **b** Plot of relaxation rate *r*_2_ *versus* Fe concentration in Fe_3_O_4_ NC/PAH/PSS hybrid nanostructures. The relaxivity value *r*_2_ was obtained from the slope of the linear fitting of the experimental data

To evaluate the potential application of Fe_3_O_4_ NC/PAH/PSS hybrid nanostructures in MRI, the proton transverse relaxation rates (1/*T*_2_) as a function of Fe ion concentration were determined using Bruker AVANCE 500WB spectrometer at 11.7 T. A linear relationship between relaxation rates with Fe ion concentration was observed, as shown in Fig. 3b. Furthermore, the transverse relaxation rates (1/*T*_2_) increased with increasing concentration of Fe_3_O_4_ NCs due to the high degree of aggregation of the Fe_3_O_4_ magnetic NPs core, demonstrating that the magnetic hybrid nanostructures could be an effective *T*_2_-weighted MRI contrast agent (Fig. 3a). Based on the slope of the plot in Fig. 3b, the transverse relaxivity value (*r*_2_) was determined to be 651.38 mM^−1^S^−1^, which is higher than that of the reported work [22]. Compared with commercial *T*_2_ contrast media, the nanoclusters can significantly improve the contrast ability of Fe after the magnetic NPs self-assembled on the basis of collective effect, thus greatly improving the angiographic effect. In the previous work, the assembled magnetite nanocrystals exhibited a higher level of saturation magnetization than individual nanocrystals due to the collective effect of magnetic nanocrystals [43, 52].

**Fig. 4:**
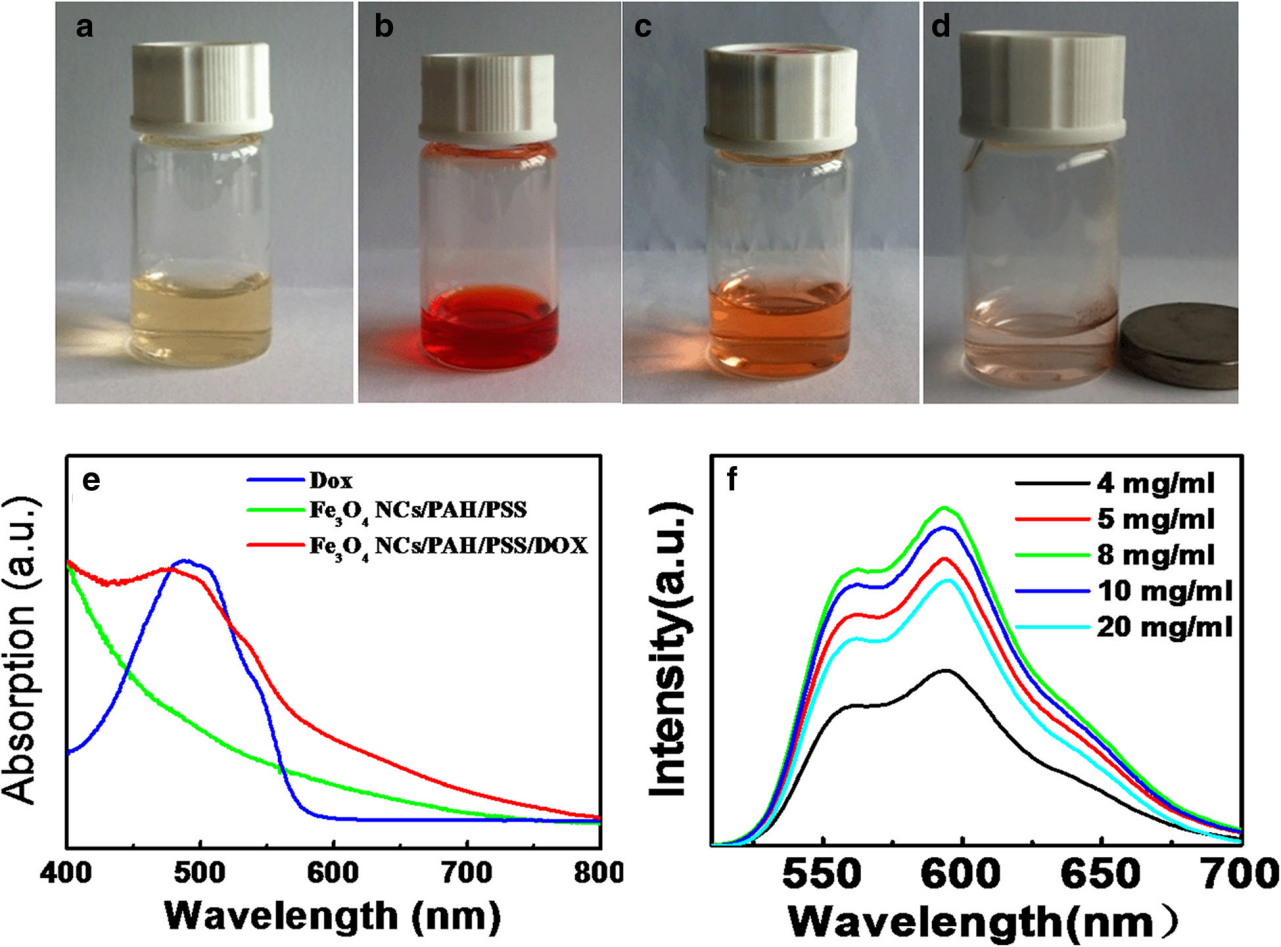
Photographs (**a**–**d**) of the different absorbed stages of DOX on the Fe_3_O_4_ NC/PAH/PSS hybrid nanostructures. The UV–vis absorption spectra (**e**) of DOX, Fe_3_O_4_ NC/PAH/PSS, and Fe_3_O_4_ NC/PAH/PSS/DOX hybrid nanostructures. The luminescence spectra (**f**) of Fe_3_O_4_ NC/PAH/PSS/DOX hybrid nanostructures when the different concentration of DOX was added into the Fe_3_O_4_ NC/PAH/PSS hybrid nanostructure solution

To evaluate the drug loading capacity of Fe_3_O_4_ NC/PAH/PSS/DOX hybrid nanostructures as drug delivery carriers, a water-soluble anticancer drug (DOX) was chosen as a model drug. The storage of DOX in the hybrid nanostructures with a high efficiency was first revealed by the color change of the solution. The color of the solution of Fe_3_O_4_ NC/PAH/PSS and the pure DOX solution was yellowish and red, respectively (Fig. 4a and b). After forming of the Fe_3_O_4_ NC/PAH/PSS/DOX hybrid nanostructures, the color of the solution became orange (Fig. 4c). Owing to the presence of Fe_3_O_4_ NPs, the DOX-loaded nanostructures in the suspension could be separated by an external magnet, suggesting that the great potential of the obtained hybrid nanostructures for magnetically targeted drug delivery (Fig. 4d). UV–vis absorption spectroscopy was used to determine the effective DOX storage capacity. Figure 4e shows the UV–vis absorption spectra of the DOX aqueous solution before and after the interaction with Fe_3_O_4_ NC/PAH/PSS hybrid nanostructures. Compared with free DOX, the similar absorption peak characteristics were observed in the Fe_3_O_4_ NC/PAH/PSS/DOX hybrid nanostructures, which is the recombination absorption peak of Fe_3_O_4_ NCs and DOX. The sample without DOX only shows the absorption peak of Fe_3_O_4_ NCs. These data indicate that DOX as a drug can be successfully absorbed onto the surface of the hybrid nanostructures. It is also found that there is an upper limit of adsorption concentration of DOX loaded on the surface of the hybrid nanostructures. Figure 4f shows the PL spectrum of the Fe_3_O_4_ NC/PAH/PSS/DOX hybrid nanostructures after centrifugation when different concentrations of DOX were added into the Fe_3_O_4_ NC/PAH/PSS solution. The luminescence intensity of DOX increases as an increase of added DOX until reaching a ceiling (8 mg/mL) with the concentration of Fe_3_O_4_ NC/PAH/PSS (1.30 × 10^−2^ mg/mL) unchanged. Afterwards, the entrapment amount decreases because of the excess DOX, which cannot be adsorbed on the surface of Fe_3_O_4_ NC/PAH/PSS/DOX hybrid nanostructures. The strongest fluorescence intensity of DOX corresponds to the concentration at 8 mg/mL, and the corresponding sample would be used for further study to perform biomedical experiment. The assured drug loading efficiency of the hybrid nanostructures is crucial for the clinical application. The loading efficiency was calculated by the area integral of DOX fluorescence intensity using the standard curve method of DOX [53, 54]. The loading efficiency was calculated up to 24.39% for the Fe_3_O_4_ NC/PAH/PSS/DOX hybrid nanostructures. Therefore, a theranostic platform has been built up based on the Fe_3_O_4_ NC/PAH/PSS/DOX hybrid nanostructures due to the effective absorption of antitumor drug DOX.

**Fig. 5:**
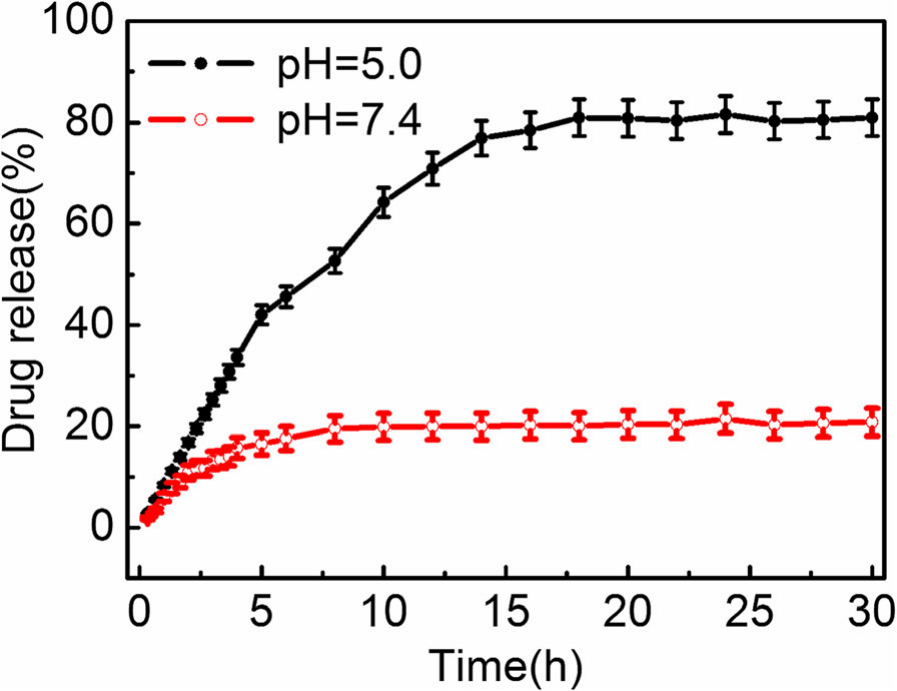
Plot of the release of DOX from the Fe_3_O_4_ NC/PAH/PSS/DOX hybrid nanostructures in PBS buffer at pH 7.4 and 5.0, respectively

The in vitro drug-releasing profiles of Fe_3_O_4_ NC/PAH/PSS/DOX hybrid nanostructures under various environmental pH values are demonstrated in Fig. 5. The Fe_3_O_4_ NC/PAH/PSS/DOX hybrid nanostructures were dialyzed through a dialysis membrane in phosphate buffers at 37 °C. The released DOX from the hybrid nanostructures was collected and then the release amount of DOX was calculated by fluorescence intensity of the supernatant. At physiological pH 7.4, the observed drug release is a slow release process. About 20 wt% of DOX was released at the initial 5 h, and then entered the stable stage of slow release. At pH 5.0, about 80 wt% of DOX was released from the hybrid nanostructures at the initial 15 h before a release plateau was reached. The plateau percentages of DOX release observed over a period of 30 h were 80 ± 3 wt% and 20 ± 3 wt% at pH 5.0 and 7.4, respectively. It can be seen that the Fe_3_O_4_ NC/PAH/PSS/DOX hybrid nanostructures have been revealed a sustained release profile and a higher DOX release rate at pH 5.0 than at pH 7.4. The low environment pH accelerates the DOX release from the Fe_3_O_4_ NC/PAH/PSS/DOX hybrid nanostructures. That is due to the protonation of the –NH_2_ group of DOX under acidic conditions, which reduces the electrostatic interaction between DOX and PSS polymers at low pH values [55]. The drug release studies indicate the good stability of electrostatically bound drug molecules in physiological pH and the triggered release at acidic conditions, similar to the reported works [56–58]. Therefore, the obtained Fe_3_O_4_ NC/PAH/PSS/DOX hybrid nanostructures are the pH-responsive systems for DOX drug delivery and suitable for the specific treatment of solid tumors [59].

**Fig. 6:**
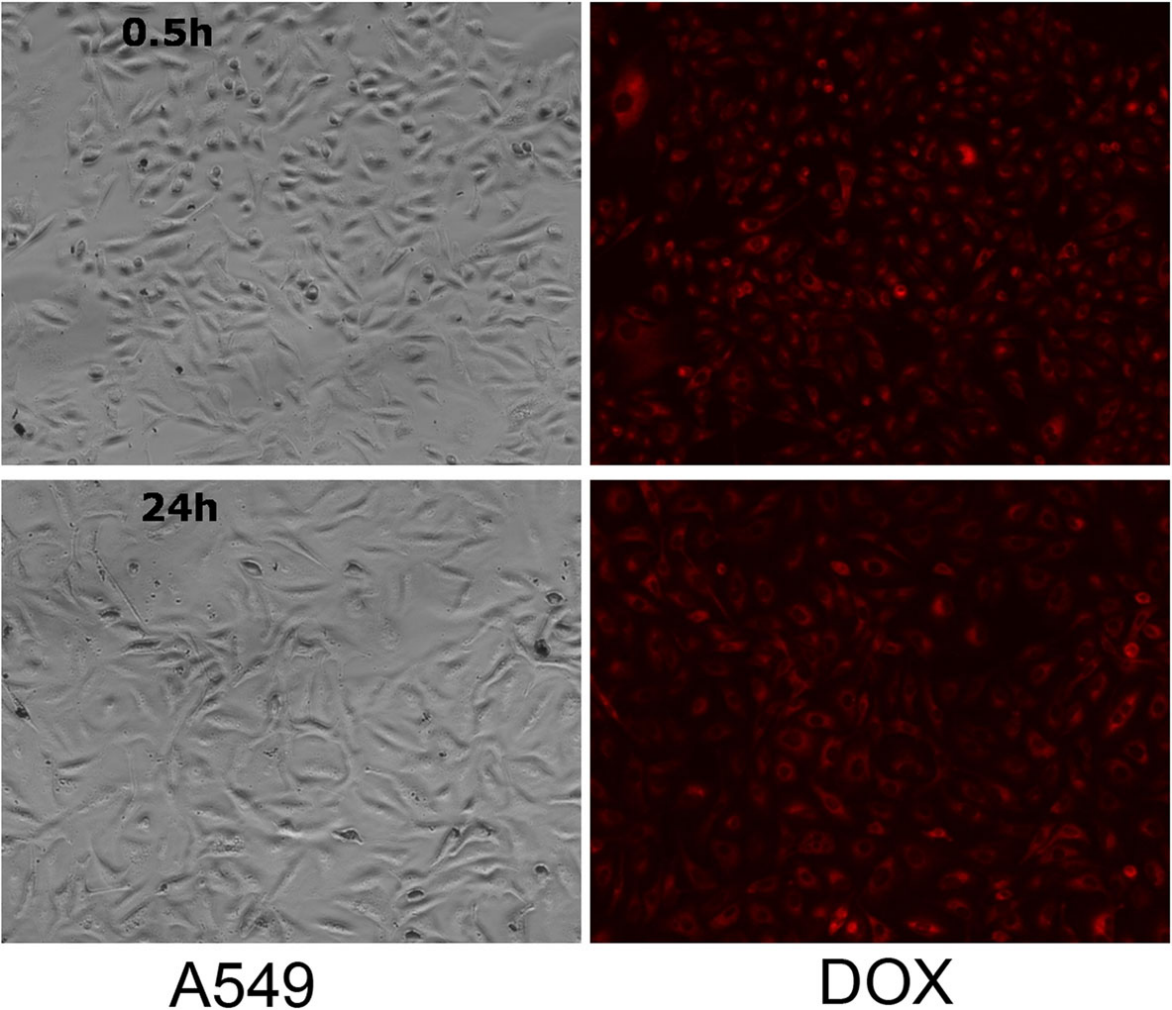
Confocal fluorescence microscopic images of A549 cells incubated with the Fe_3_O_4_ NC/PAH/PSS/DOX hybrid nanostructures at 37 °C for **a** 0.5 h and **b** 24 h. Scale bar, 20 μm

The cellular uptake and cytotoxicity are key factors to evaluate the potential of a new drug delivery system. The cellular uptake and cytotoxicity of Fe_3_O_4_ NC/PAH/PSS/DOX hybrid nanostructures on A549 cell lines were studied. The intercellular uptake of Fe_3_O_4_ NC/PAH/PSS/DOX hybrid nanostructures was investigated using optical and fluorescence microscopy, which was mainly realized by monitoring the fluorescence from DOX. The Fe_3_O_4_ NC/PAH/PSS/DOX hybrid nanostructures have been proved to be effective in delivering DOX to cancer cells. As shown in Fig. 6, strong red fluorescence from DOX was observed in cancer cells after incubation for 24 h. The hybrid nanostructures were internalized mainly through endocytosis [60]. After cell uptake, the hybrid nanostructures released DOX in the acidic environment around the endosome/lysosomes, in which a sufficiently low pH (4.3) could trigger an effective DOX release (pH 5.0, Fig. 5). The Fe_3_O_4_ NC/PAH/PSS/DOX hybrid nanostructures exhibited time-dependent uptake in A549 cancer cells, as shown in Fig. 6. At 0.5 h post incubation, red fluorescence was visible around the cells. The results show that the hybrid nanostructures containing DOX mainly stayed around the A549 cells. However, when the incubation time increased to 24 h, the intercellular fluorescence signal increased from the A549 cells. Obviously, many hybrid nanostructures can enter the cancer cells over time. These results confirm that the Fe_3_O_4_ NC/PAH/PSS/DOX hybrid nanostructures can efficiently transfer DOX into A549 cells. DOX released from the hybrid nanostructures in the cytoplasm passes through the nuclear membrane and eventually accumulates in the nucleus, killing cells by causing changes in DNA conformation [61].

**Fig. 7:**
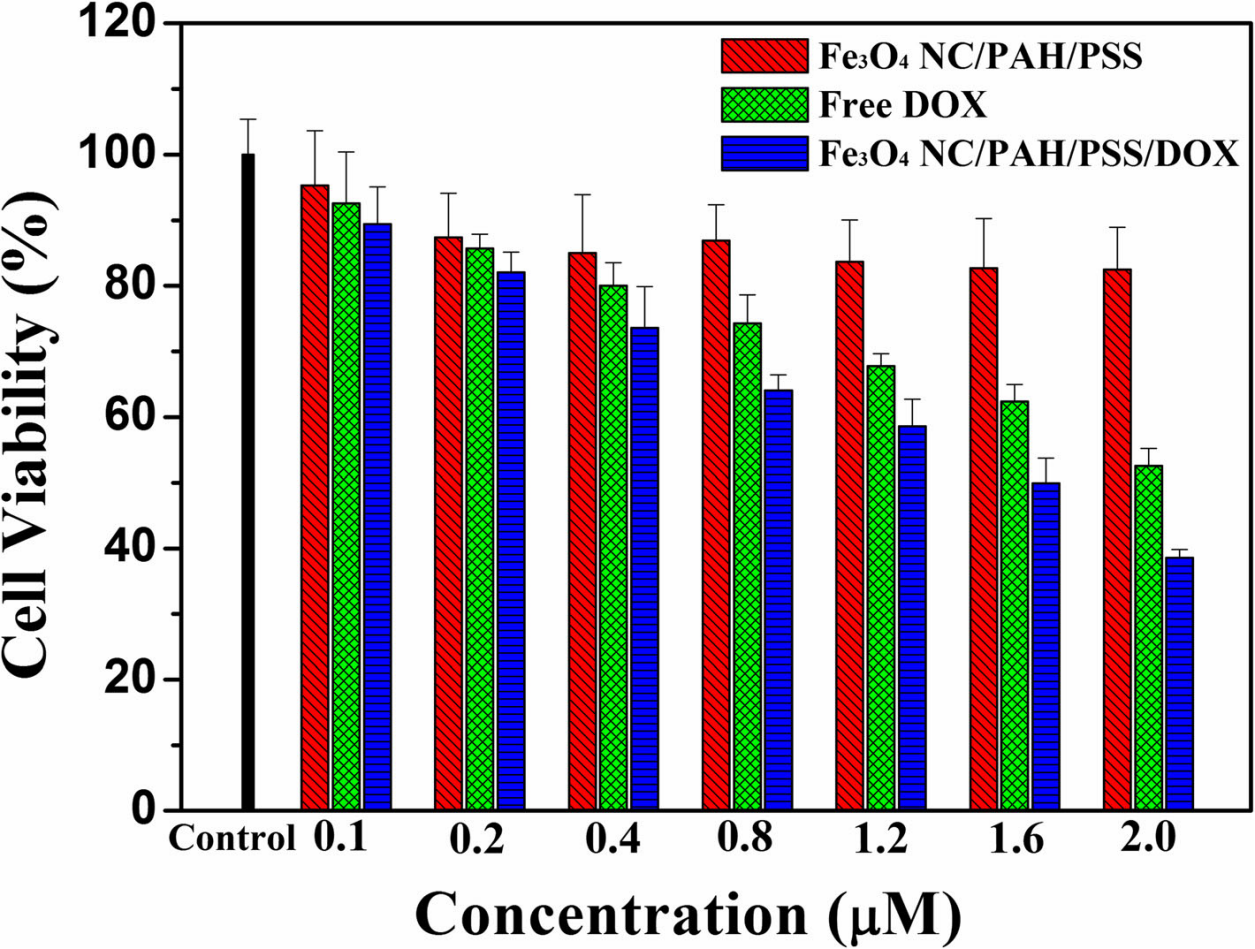
Relative viability of A549 cells incubated with free DOX, Fe_3_O_4_ NC/PAH/PSS, and Fe_3_O_4_ NC/PAH/PSS/DOX hybrid nanostructures at different concentrations for 24 h. Error bars were based on triplicate samples

In order to evaluate the pharmacological activity of the Fe_3_O_4_ NC/PAH/PSS/DOX hybrid nanostructures, the cytotoxicity to A549 cells in vitro was determined by MTT method. Figure 7 shows the cell activity of free DOX, Fe_3_O_4_ NC/PAH/PSS, and Fe_3_O_4_ NC/PAH/PSS/DOX hybrid nanostructures with different concentrations after incubation with A549 cells for 24 h. The material amounts were calculated according to the concentration of DOX. The free DOX concentration was the same as the DOX concentration in Fe_3_O_4_ NC/PAH/PSS/DOX hybrid nanostructures, and the concentration of Fe_3_O_4_ NC/PAH/PSS hybrid nanostructure was the same as the Fe_3_O_4_ NC/PAH/PSS concentration in the Fe_3_O_4_ NC/PAH/PSS/DOX hybrid nanostructures. Each sample was cultured with A549 cells for 24 h. The concentration of Fe_3_O_4_ NC/PAH/PSS ranged from 0.1 to 2.0 μΜ, and the cell survival rate exceeded 85%. This indicated that Fe_3_O_4_ NC/PAH/PSS hybrid nanostructures had no obvious cytotoxicity to cancer cells and had good biocompatibility. After incubating with cancer cells for 24 h, however, the free DOX and Fe_3_O_4_ NC/PAH/PSS/DOX hybrid nanostructures showed obvious cytotoxicity. The cellular viability progressively decreased with increasing effective DOX concentration. As shown in Fig. 7, when the effective DOX concentration was increased from 0.1 up to 2.0 μM, the relative cell viability decreased from about 92% to about 50% for free DOX, and from about 89% to about 40 % for Fe_3_O_4_ NC/PAH/PSS/DOX hybrid nanostructures, respectively.

These results indicate that both free DOX and Fe_3_O_4_ NC/PAH/PSS/DOX hybrid nanostructures have dose-dependent cytotoxicity to cancer cells. The cytotoxicity originates from the loaded DOX rather than Fe_3_O_4_ NC/PAH/PSS hybrid nanostructures. Cell uptake of free DOX is faster than that of DOX-loaded hybrid nanostructures. This reason is that small DOX molecules can quickly spread into cells, while Fe_3_O_4_ NC/PAH/PSS/DOX hybrid nanostructures must be endocytosis in order to enter cancer cells. Because of the hypoxia-induced coordinated upregulation of glycolysis, the acidic extracellular environment of solid tumors is stronger than that of normal tissues [62]. At the cellular level, the internalization of most of the hybrid nanostructures will take place through endocytosis. With the increase of DOX concentration, more and more hybrid nanostructures loaded with DOX are endocytosed into cancer cells. After cellular endocytosis, the DOX-loaded hybrid nanostructures usually enter the early endosomes, then enter the late endosomes/lysosomes, and finally fused with lysosomes. Furthermore, both endosomes (pH 5.0–6.0) and lysosomes (pH 4.5–5.0) have an acidic microenvironment. In our study, the pH-responsive Fe_3_O_4_ NC/PAH/PSS/DOX hybrid nanostructures were more likely to decompose and release drugs in acidic environments, thus effectively reducing side effects, prolonging half-life of drugs, and providing more effective and lasting treatment. Due to the main target of DOX being cell nucleus, DOX can bind to double-stranded DNA to form DNA adducts, inhibit the activity of topoisomerase and induce cell death (apoptosis) [63]. As a result, the released DOX molecules were located in the cell nucleus. Therefore, the obtained Fe_3_O_4_ NC/PAH/PSS/DOX hybrid nanostructures may have good potential for cancer chemotherapy.

**Fig. 8:**
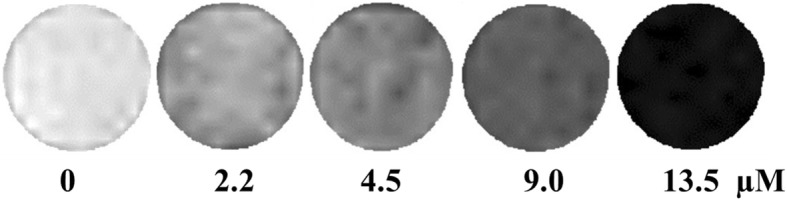
*T*_2_-weighted cellular MR images of A549 cells incubated with the Fe_3_O_4_ NC/PAH/PSS/DOX hybrid nanostructures at a Fe concentration of 2.2, 4.5, 9.0, and 13.5 μM, respectively

As discussed above, the Fe_3_O_4_ NC/PAH/PSS/DOX hybrid nanostructures exhibit high relaxivity in aqueous solution and can be uptaken efficiently by A549 cells. The intracellular MRI of the Fe_3_O_4_ NC/PAH/PSS/DOX hybrid nanostructures were then investigated by incubation of A549 cells with the hybrid nanostructures with different Fe_3_O_4_ concentrations. Figure 8 presents the *T*_2_-weighted MRI of A549 cells. With the increase of Fe_3_O_4_ concentration in Fe_3_O_4_ NC/PAH/PSS/DOX hybrid nanostructures, the cellular MRI signal increased gradually (Fig. 8). Currently, cell labeling is mainly accomplished by the endocytosis of Fe_3_O_4_ NC/PAH/PSS/DOX hybrid nanostructures as *T*_2_-negative contrast agents. These results demonstrate that the Fe_3_O_4_ NC/PAH/PSS/DOX hybrid nanostructures can be internalized into cells and exhibit good *T*_2_-weighted MRI contrast for cellular imaging. Our current research is limited to the cellular level. Future in vivo studies would be necessary for the practical application of the Fe_3_O_4_ NC/PAH/PSS/DOX hybrid nanostructures. To specially target a specific site in animal studies, small ligands such as lactic acid and folic acid (both containing carboxyl groups) would require to be used to conjugate amino-terminated Fe_3_O_4_ NC/PAH/PSS/DOX hybrid nanostructures.

## Conclusion

The multifunctional Fe_3_O_4_ NC/PAH/PSS/DOX hybrid nanostructures were developed as the pH-triggered drug delivery system for effective cancer chemotherapy and MRI. The quasi-spherical Fe_3_O_4_ NCs can significantly improve the contrast ability of MRI compared with Fe_3_O_4_ NPs. The Fe_3_O_4_ NC/PAH/PSS/DOX hybrid nanostructures can act as contrast agents to enhance MRI and as a fluorescence probe for cell imaging. The DOX can be released from the Fe_3_O_4_ NC/PAH/PSS/DOX hybrid nanostructures at acidic environment and exhibit an excellent cellular cytotoxic effect on A549 cells. The Fe_3_O_4_ NC/PAH/PSS/DOX hybrid nanostructures as multifunctional theranostic platform have great potential for biomedical application, including MRI, fluorescence imaging, and stimuli-responsive drug delivery nanocarriers.

## Additional file

Additional file 1: **Figure S1.** TEM image of Fe_3_O_4_ nanoparticles. **Figure S2.** Zeta potential at the different synthesis stages of Fe_3_O_4_ NC/PAH/PSS/DOX hybrid nanostructures as theranostic agents for magnetic resonance imaging and drug delivery. (DOCX 399 kb)

## Data Availability

Data sharing is not applicable to this article as no datasets were generated or analyzed during the current study. Please contact the author for data requests.

## Abbreviations

1/*T*_2_: The proton transverse relaxation rates
A549: Human lung cancer
DLS: Dynamic light scattering
DMSO: Dimethyl sulfoxide
DOX: Doxorubicin hydrochloride
FBS: Fetal bovine serum
Fe_3_O_4_: Iron oxide
FOV: Field of view
LBL: Layer-by-layer
MRI: Magnetic resonance imaging
MTT: Methyl thiazolyltetrazolium
NA: Number of averages
NaOA: Sodium oleate
NaOH: Sodium hydroxide
NC: Nanocluster
NCs: Nanoclusters
NH_4_F: Ammonium fluoride
NPs: Nanoparticles
OA: Oleic acid
OD: Optical density
ODE: 1-Octadecene
PAH: Poly(allylamine hydrochloride)
PSS: Poly(sodium 4-styrenesulfonate)
S/O/W: Solid-in-oil-in-water
SDBS: Sodium dodecyl benzene sulfonate
TE: Echo time
TEM: Transmission electron microscope
TR: Repetition time
