# Supplementary material for: Fabrication of Hybrid Nanostructures Based on Fe3O4 Nanoclusters as Theranostic Agents for Magnetic Resonance Imaging and Drug Delivery
Source: Nanoscale Res Lett. 2019 Jun 7;14:200. doi: 10.1186/s11671-019-3026-7 (PMC6555842; doi:10.1186/s11671-019-3026-7)
Supplement: Supplementary file 1 — Figure S1. TEM image of Fe3O4 nanoparticles. Figure S2. Zeta potential at the different synthesis stages of Fe3O4 NC/PAH/PSS/DOX hybrid nanostructures as theranostic agents for magnetic resonance imaging and drug delivery. (DOCX 399 kb) [file 11671_2019_3026_MOESM1_ESM.docx]

**Supplementary Materials**

**Fabrication of Hybrid Nanostructures based on Fe_3_O_4_ Nanoclusters as Theranostic Agents for Magnatic Resonance Imaging and Drug Delivery**

Junwei Zhao^†2,4^, Xiang Li^†3,4^, Xin Wang^3^ & Xin Wang^*1,4^

^1^ Henan Key Laboratory of Photovoltaic Materials, Henan University, Kaifeng 475004, P. R. China; xwang2008@vip.henu.edu.cn (X. W.)

^2^ Materials Science and Engineering School& Henan Key Laboratory of Special Protective Materials, Luoyang Institute of Science and Technology, Luoyang 471023, P. R. China; jwzhao2010@163.com (J. Z.)

^3^ College of Materials Science and Engineering, Jilin University, Changchun 130022, P. R. China; wang-xin@jlu.edu.cn (X. W.)

^4^ Division of Nanobiomedicine, Suzhou Institute of Nano-Tech and Nano-Bionics, Chinese Academy of Sciences, Suzhou 215123, P. R. China; xli2013@sinano.ac.cn (X.L.)

*Correspondence and requests for materials should be addressed to X.W. (email: xwang2008@vip.henu.edu.cn)

^†^These authors contributed equally.

**
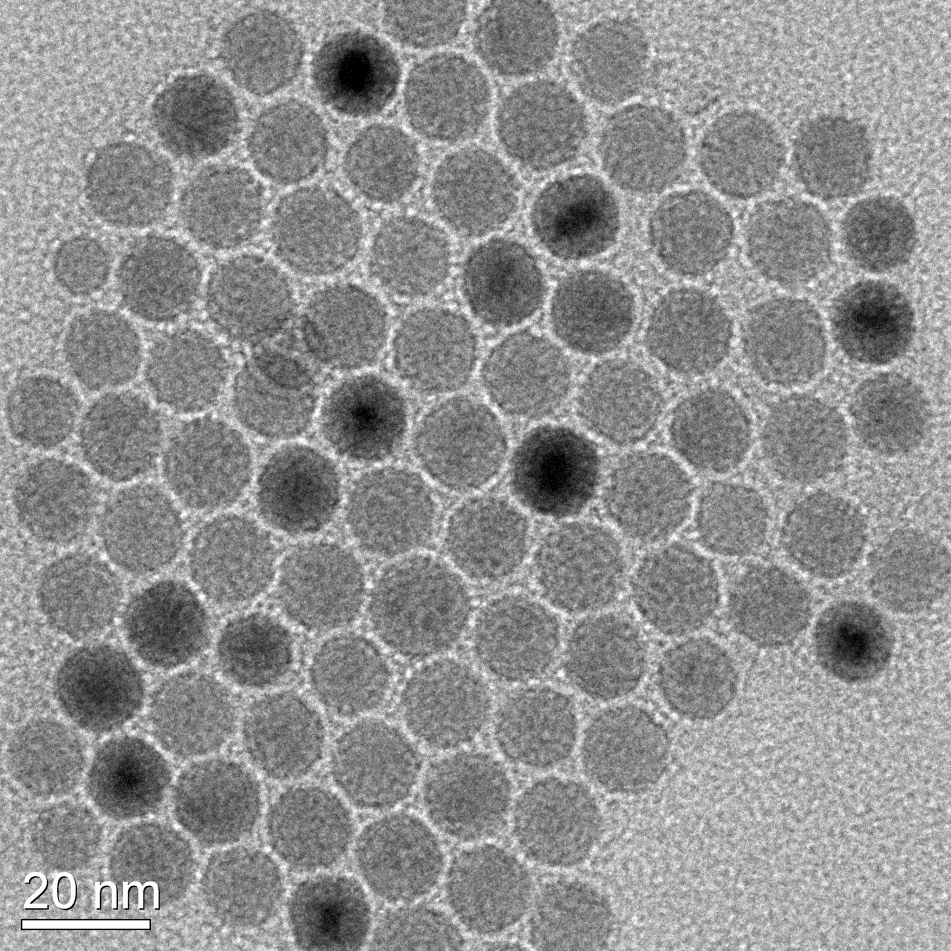
**

Figure S1. TEM image of Fe_3_O_4_ nanoparticles.

**
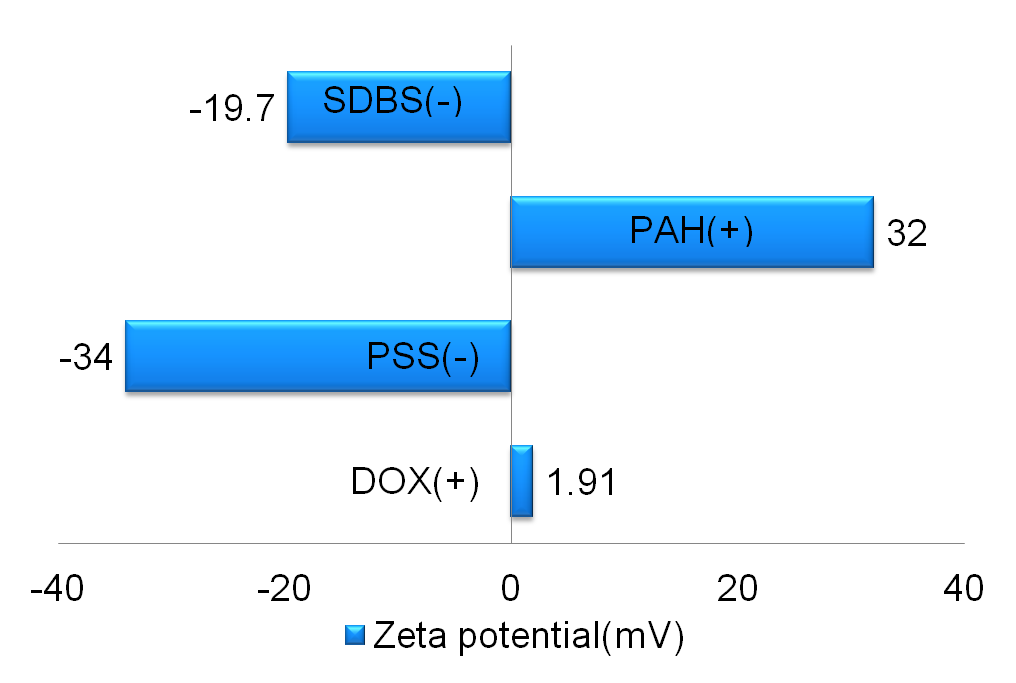
**

Figure S2. Zeta pontentialat the different synthesis stages of Fe_3_O_4_ NC/PAH/PSS/DOX hybrid nanostructures as theranotics agents for magnatic resonance imaging and drug delivery.
